# PRELP-associated transcriptomic remodeling links alternative splicing and immune checkpoint blockade outcome in melanoma

**DOI:** 10.3389/fimmu.2026.1896231

**Published:** 2026-09-15

**Authors:** Karthikeyan Subbarayan, Kamatchi Ulagappan, Mario Udinotti, Dimitrios Kokoretsis, Claudia Wickenhauser, Barbara Seliger

**Affiliations:** 1 Institute of Translational Immunology and Faculty of Health Sciences, Brandenburg Medical School “Theodor Fontane”, Brandenburg an der Havel, Germany; 2 Institute of Pathology, Section Immunopathology, Martin Luther University Halle-Wittenberg, Halle, Germany; 3 Fraunhofer Institute for Cell Therapy and Immunology (IZI), Leipzig, Germany

**Keywords:** alternative splicing, exon skipping, immune checkpoint blockade, interferon response, PRELP, ScRNA-seq

## Abstract

**Background:**

Cutaneous melanoma is an aggressive malignancy characterized by a high metastatic potential and variable response to immune checkpoint blockade (ICB). The extracellular matrix protein proline/arginine-rich end leucine-rich repeat protein (PRELP), an extracellular matrix protein, contributes to stromal architecture and tumor–immune interactions. This study investigated whether PRELP expression drives transcriptomic remodeling, alternative splicing, interferon (IFN)-related signaling pathways to influence immunotherapy responsiveness in melanoma.

**Methods:**

Bulk RNA sequencing was performed on PRELP-high and PRELP-low BUF1088 melanoma cells to assess differential gene expression and alternative splicing landscapes. Downstream pathway changes were characterized using hallmark enrichment analysis. The translational relevance of the profiles was assessed using publicly available single-cell RNA sequencing datasets and clinical melanoma ICB cohorts.

**Results:**

Differential splicing analysis identified 274 significant splicing events across 230 genes, predominantly exon skipping events. Key differentially spliced genes included AAR2, SACM1L, CD22, ACTB, MYO5A, WARS1, BTN3A3, IL24, and HNRNPA3. In parallel, PRELP-high melanoma cells showed enrichment of IFN-gamma and IFN-alpha response pathways accompanied by increased expression of IFI27, IFI44, IRF7, and IFITM3. In publicly available clinical datasets, PRELP expression and a composite PRELP-related gene signature, (PRELP, IRF7, IFI27, IFI27L1, IFI44, IFITM3, WARS, and IL24) were significantly enriched in ICB responders versus non-responders. Single-cell mapping localized endogenous PRELP expression to specific stromal subsets, primarily extracellular matrix-remodeling myofibroblastic CAFs (myCAFs) and IFN-responsive CAFs.

**Conclusion:**

These findings demonstrate that PRELP expression in melanoma orchestrates extensive transcriptomic remodeling, linking stromal matrix programs with IFN-associated antitumor signaling and alternative splicing landscapes. Consequently, PRELP-associated expression and splicing signatures represent promising predictive biomarkers to guide patient selection for immunotherapy response.

## Introduction

Cutaneous melanoma represents one of the most common immunogenic human malignancies and has become a central model for the development of cancer immunotherapy, yet it remains equally paradigmatic for therapeutic heterogeneity (1, 2). Although immune checkpoint blockade (ICB) has transformed outcomes for a subset of patients, primary and acquired resistance of melanoma remain major clinical barriers. Consequently, the identification of molecular and microenvironmental determinants that govern melanoma immune responsiveness remains a premier challenge in tumor immunology. Accumulating evidence indicates that successful immunotherapy depends not only on tumor antigenicity, but also on the preservation of inflammatory signaling, antigen presentation machinery (APM) and tumor cell responsiveness to immune-mediated pressure (1, 3). Genomic analyses of responses to cytotoxic T lymphocyte-associated protein-4 (CTLA-4) blockade, pretreatment transcriptomic studies of anti-programmed cell death protein-1 (PD-1) therapy, and longitudinal analyses of relapse after pembrolizumab have demonstrated that durable clinical benefit requires coordinated immune activation within the tumor microenvironment (TME), enabling tumor cells to remain responsive to immune effector cues (1, 2, 4).

Single-cell and spatially resolved studies have further refined this perspective by showing that melanoma is not simply a disease of malignant cells, but rather a dynamic highly organized multicellular ecosystem (5). Early single cell analyses revealed that metastatic melanoma consists of transcriptionally diverse malignant, immune, stromal, and endothelial cell populations, while subsequent studies by Jerby-Arnon and colleagues identified malignant cell resistance programs associated with T cell exclusion and resistance to ICB (6). In parallel, Sade-Feldman and colleagues identified distinct intratumoral CD8+ T cell states associated with patient outcome after checkpoint immunotherapy (5, 7). These studies collectively support the concept that successful therapy depends on coordinated interaction between tumor cell programs, immune cell state, and stromal context rather than on a single molecular feature.

Among stromal determinants of tumor immunity, cancer-associated fibroblasts (CAFs) act as key regulators due to their marked functional heterogeneity and their ability to either facilitate or constrain antitumor immunity (8, 9). Initially characterized in pancreatic cancer, through the identification of myofibroblastic and inflammatory CAF subtypes the CAF framework has expanded on the spatial context including antigen-presenting and other context-dependent subsets across multiple cancer types. Recent studies in skin cancer have confirmed the presence of unique matrix-rich and immunomodulatory CAF programs within the TME (9, 10). In murine melanoma models, stromal populations also undergo dynamic temporal changes during tumor progression, highlighting fibroblasts as active regulators of tumor evolution and immune modulation rather than passive structural components (11).

Within this broader stromal and immune landscape, the proline/arginine-rich and leucin-rich repeat protein (PRELP) has emerged as a molecular link at the intersection of stromal remodeling and immune activity (12, 13). PRELP is a family member of the small leucine-rich proteoglycans that interacts with perlecan and collagen networks and has implicated in functions beyond structural anchoring, including the inhibition of complement membrane attack complex formation and modulation of transforming growth factor (TGF)-β signaling (12–14). In melanoma, reduced PRELP expression correlates with decreased expression of the major histocompatibility complex (MHC) class I antigen processing component, impaired IFN-γ pathway activity, lower T cell infiltration, and highly aggressive tumor phenotypes. Conversely, PRELP overexpression increased MHC class I and the T cell-recruiting chemokine CCL5 in melanoma cells (15). Together, these findings imply that PRELP levels alter both the extracellular matrix (ECM) architecture and tumor immunogenicity (15).

An additional and increasingly important layer of melanoma biology involves post-transcriptional regulation (16, 17). Alternative splicing (AS) and intron retention influence mesenchymal cell states, cytolytic activity, resistance to MAPK-targeted therapy, and the generation of shared splicing-derived neoantigens with potential relevance to immunotherapy (16, 18, 19). These data imply that melanoma cell states are defined not only by gene expression abundance but also by transcript structural diversity. However, the mechanistic connections between RNA splicing programs, PRELP-centered ECM biology and fibroblast-state heterogeneity remains poorly understood (10, 15, 16, 19).

To address this knowledge gap, we investigated whether PRELP coordinates a distinct melanoma state, where ECM architecture, fibroblast heterogeneity, IFN pathways and RNA processing converge to determine checkpoint sensitivity. We hypothesized that PRELP-high melanoma states display unique alternative-splicing profiles, enhanced IFN response and antigen presentation-associated programs, and an immune-supportive stromal microenvironment compared with PRELP-low states. To address this, we applied an integrative analytical framework combining bulk RNA sequencing (RNA-seq), differential splicing analysis, pathway enrichment, public single-cell RNA-seq (scRNA-seq) mapping and clinical validation across public checkpoint response cohorts.

## Methods

### Cell culture and transfection

The melanoma cell lines used in this study were either provided by Dr. Soldano Ferrone (Harvard University, Boston, MA, USA) or obtained from the European Searchable Tumor Cell Line and Data Bank, ESTDAB. The BJ fibroblast cell line was purchased from American Type Culture Collection (ATCC) (Manassas, USA). Melanoma cells were cultured in Roswell Park Memorial Institute 1640 medium (RPMI1640, Invitrogen, Carlsbad, CA, USA), the BJ fibroblast cell line in Eagle’s minimum essential medium (EMEM) supplemented with 10% fetal bovine serum (FBS) (Gibco, Waltham, USA), 1% L-glutamine (Sigma-Aldrich, Burlington, USA) and 1% penicillin-streptomycin (Sigma-Aldrich). Adherent cells were detached using trypsin-EDTA (0.25%) (Gibco, Waltham, USA). Cell cultures were maintained in T75 flasks in a humidified incubator at 37 °C with 5% CO2.

For transfection, BUF1088, BUF1102 and BUF537 melanoma cells were grown to approximately 70–80% confluence and transfected with either a PRELP expression vector (PRELP-high) or the corresponding mock vector control (PRELP-low) using Effectene Transfection Reagent (Qiagen, Hilden, Germany) for 48 hours, before the cells were harvested and subjected to downstream analysis.

### RNA extraction, quality analysis, and bulk RNA sequencing

Total cellular mRNA was isolated from PRELP-high and PRELP-low melanoma cells using RNA isolation kit (Machery-Nagel, Düren, Germany) according to the manufacturers’ protocol. Agilent 2100 bioanalyzer (Agilent, Santa Clara, USA) was used to assess the quality and concentration of RNA. For bulk RNA sequencing (RNA-seq), strand-specific libraries were generated by Novogene (Novogene, Beijing, China) following ribosomal RNA depletion. The purified RNA was fragmented (250–300 bp) and used as template for random primed first-strand cDNA synthesis, followed by second-strand cDNA synthesis incorporating dUTPs. Double-stranded cDNA fragments underwent end repair, A-tailing and adapter ligation. cDNAs around 350~400 bp were selected using AMPure XP beads and the dUTP-containing second strand was degraded using USER enzyme prior to final PCR amplification and sequencing.

### Alternative splicing analysis and transcript validation

Alternative splicing (AS) events were identified and quantified using the rMATS (4.1.2) software (20), which categorizes AS events into 5 categories and enables differential AS analyses in samples with biological replicates: skipped exons (SE), mutually exclusive exons (MXE), alternative 5′ splice sites (A5SS), alternative 3′ splice sites (A3SS), and retained introns (RI). Isoform counts were normalized by effective length, and the ratio of exon inclusion to exon skipping was computed to evaluate differential splicing between groups.

For validation, total RNA was reverse-transcribed into cDNA using the ReverseAid First Strand cDNA Synthesis Kit according to the manufacturer’s protocol (Thermo Fisher Scientific, Waltham, MA, USA). Differentially spliced transcripts identified by rMATS were selected for validation by reverse-transcription PCR using primers designed in the flanking constitutive exons to distinguish exon-included and exon-skipped isoforms. PCR products were resolved on agarose gels, visualized by nucleic acid staining, and quantified by densitometric analysis using iBright750 Imaging System (Invitrogen, Waltham, USA). Band intensities were background-subtracted and corrected for amplicon length. Percent spliced-in (PSI) values were calculated as the corrected exon-included isoform signal divided by the sum of corrected exon-included and exon-skipped isoform signals. The metric ΔPSI was calculated by subtracting PSI of vector-control cells from the PSI of PRELP-high cells.

For quantitative gene-expression analysis, cDNA was subjected to real-time PCR using a Bio-Rad CFX real-time PCR system and SYBR Green PCR Master Mix according to the manufacturer’s instructions (Promega, Madison, WI, USA). Data are available as mean ± SEM of at least three independent experiments. The primer sequences used in this study are listed in **Supplementary Table 1**.

### Pathway enrichment analysis and immunoblotting

Hallmark gene sets from the Molecular Signatures Database (MSigDB) were obtained via msigdbr, and over-representation analysis of upregulated genes was performed using the clusterProfiler R package applying Benjamini-Hochberg FDR corrections.

For protein analysis, total cellular proteins were extracted, and protein concentrations quantified using the Piece BCA protein kit (Thermo Fisher Scientific). Equal amounts of protein (10–30 µg/lane) were separated by gel electrophoresis via SDS-PAGE and transferred onto nitrocellulose membranes (Invitrogen) using an iBlot2 transfer system (Invitrogen). The blots were blocked and incubated overnight at 4 °C with primary antibodies (Ab) against IFI27, STAT1, pSTAT1 (Cell Signaling Technology, Danvers, USA) and TAP1 (Sigma-Aldrich), followed by incubation with the appropriate horseradish peroxidase (HRP)-conjugated secondary Abs (Cell Signaling Technology) for 1 hour at room temperature. Protein bands were visualized using an enhanced chemiluminescence (ECL) detection system (Cell signaling Technology).

### *In vitro* fibroblast conditioning and phenotyping

BJ fibroblasts (1 X 10^6^ cells) were cultured for 6 days under three distinct conditions: unconditioned RPMI medium (BJ control), 10% (v/v) conditioned medium from melanoma cells transfected with an empty-vector control (BJ vector CM), or 10% (v/v) conditioned medium from melanoma cells transfected with a PRELP-overexpression plasmid (BJ PRELP CM). Media were renewed three times during the 6-day culture period across three biological replicates.

Fibroblasts were then harvested for flow cytometry. After exclusion of doublets, myofibroblastic CAFs were isolated by sequential gating for FAP^+^TEM8^+^ cells and αSMA^+^CD29^+^ markers. The resulting myCAF population was separated into CD9^-^ and CD9^+^ fractions and tracking markers including LAMP5 and Syndecan-1/CD138 expression were used to define specialized sub lineages, such as ECM-, TGFβ-, IFNαβ-, Acto-, and wound-myCAF subpopulations. Frequencies of the total myCAF population were calculated relative to the total fibroblast population, whereas individual myCAF subpopulations were quantified relative to their respective parent gates.

### Flow cytometry

For determination of the human leukocyte antigen class I (HLA-I) surface expression, 1 × 10^5^-1 × 10^6^ cells were stained with Abs against HLA-I (HLA-ABC) (Invitrogen, Waltham, USA) for 15 min at room temperature, washed with PBS and analyzed using a Navios 3L10C flow cytometer (Beckman Coulter GmbH, Krefeld, Germany). Data were evaluated with the Kaluza (Beckman Coulter) analysis software and are presented as mean specific fluorescence intensity (MFI).

### Clinical cohort evaluation

To assess the predictive performance of candidate gene signatures, processed transcriptomic profiles from three independent melanoma immunotherapy cohorts available through the TIGER portal were analyzed (21): (i) the GSE115821 dataset, comprising patients treated with anti-CTLA-4, anti-PD-1, or combination therapy (22, 23); (ii) an isolated anti-CTLA-4 clinical cohort (24); and (iii) the PRJEB23709 dataset, consisting of patients receiving anti-PD-1 monotherapy or combined anti-PD-1/anti-CTLA-4 treatment (25). Differential gene expression profiles were compared between clinical responders and non-responders across all cohorts (21, 26).

### Single-cell RNA sequencing analysis

Public melanoma scRNA-seq datasets GSE115978 and GSE72056 were analyzed to evaluate the cellular distribution of PRELP and selected IFN-related genes. and retrieved form the Gene Expression Omnibus (GEO). Matrices were normalized via log1p(TPM) shifts, where appropriate, and downstream processing, Uniform Manifold Approximation and Projection (UMAP) visualizations, and expression dot blots were generated using the Seurat framework.

### Statistical analysis

Graphical visualization and statistical analyses were performed using Microsoft Excel (Office 365), BioRender, and R version v4.5.1. Splicing shifts were verified with rMATS v4.1.2 with biological replicates. Hallmark pathway enrichment analysis was performed using clusterProfiler with Benjamini-Hochberg false discovery rate correction. Adjusted p-values or false discovery rate (FDR) threshold of < 0.05 were considered statistically significant.

## Results

### Transcriptomic sequencing metrics

To characterize the molecular network regulated by PRELP, bulk RNA-seq libraries were constructed from PRELP-high and PRELP-low BUF1088 melanoma cells. Sequencing yielded a total of 381, 826, 360 raw reads across six samples. Following quality filtering and trimming, including assessment of sequencing error rates and GC-content distribution, 333, 457, 554 clean reads were retained, representing an 87.3% efficiency rate (**Table 1**). Genomic mapping against *Homo sapiens* reference assembly (Ensembl release 109, GRCh38 primary assembly) using the HISAT2 showed that approximately 85% of total clean reads mapped uniquely to a single genomic location with highly structural uniformity between PRELP-low and PRELP-high groups. These results indicate that the RNA-seq datasets were of good quality and suitable for downstream transcriptomic and AS analyses.

**Table 1:** High throughput RNA-seq sequencing and alignment metrics.

Samples	Total reads	Clean reads	Unique mapped	Reads filter %	Unique mapped %
PRELP-low-1	63288180	55252031	53826293	87.30%	85.05%
PRELP-low-2	62281092	54541365	53163317	87.57%	85.36%
PRELP-low-3	61455502	53438119	52030298	86.95%	84.66%
PRELP-high-1	65249200	57164837	55774640	87.61%	85.48%
PRELP-high-2	66717594	58292894	56905023	87.37%	85.29%
PRELP-high-3	62834792	54768308	53442803	87.16%	85.05%

Summar of RNA-seq quality metrics and alignment efficacies for PRELP-high (PRELP transfected) and PRELP-low (vector-control) BUF1088 melanoma cells. Data are reported for individual biological replicates (n=3 per group). Metrics include total sequencing reads, clean reads following filtering, uniquely mapped reads, read retention rate (%), and unique alignment percentage (%) mapped against the human reference sample.

### Distribution of mapped sequencing reads across genomic regions

Analysis of the genomic distribution of mapped RNA-seq reads confirmed that the libraries from both PRELP-low and PRELP-high BUF1088 melanoma cells consisted primarily of mature coding transcripts (**Figure 1**). Across all six libraries, exonic reads accounted for approximately 95.05% to 96.23% of the total aligned reads, whereas intronic and intergenic regions represented only 2.96% to 3.98% and 0.81% to 0.96%, respectively. This alignment profile confirmed the quality and purity of the isolated RNA validating its use for downstream splicing and transcriptomic analysis.

**Figure 1:**
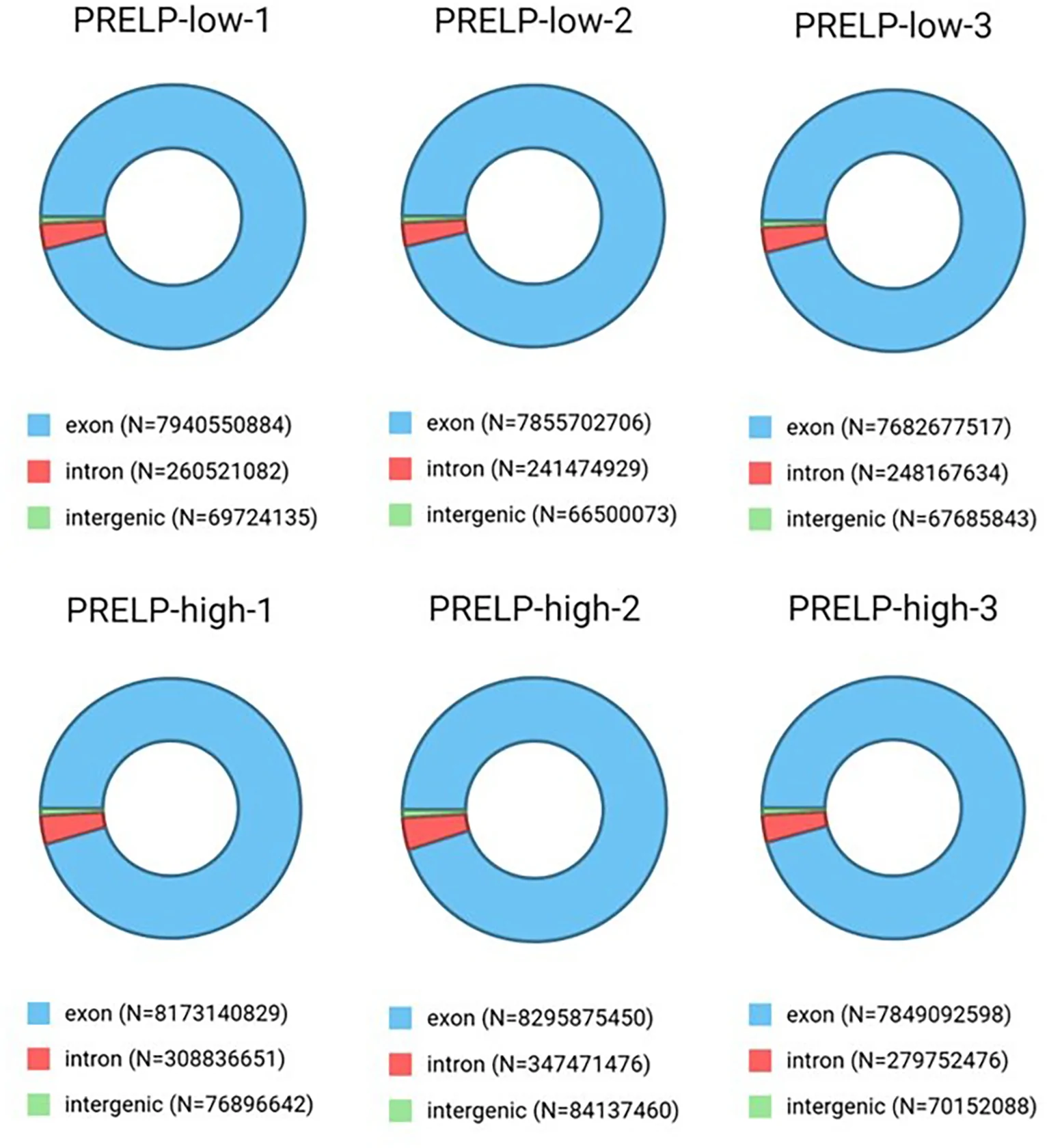
PRELP expression alters genomic distribution of mapped RNA-seq reads in 1088 melanoma cells. Quantification of mapped RNA-seq signal proportions assigned to exonic, intronic, and intergenic regions across biological replicates of BUF1088 melanoma cells. Pie charts display the transcriptomic distribution for PRELP-low vector-control cells (n = 3 independent biological replicates) and PRELP-high transfected cells (n = 3). Percentages reflect the proportion of total uniquely mapped reads aligning to each annotated genomic feature based on the standard reference genome annotation.

### Alternative splicing profiles and top splicing targets in PRELP-high and PRELP-low melanoma cells

Differential splicing analysis identified five distinct categories of AS events: skipped exon (SE), mutually exclusive exon (MXE), alternative 5′ splice site (A5SS), alternative 3′ splice site (A3SS), and retained intron (RI) (**Figure 2**). In total, 94, 778 alternative splicing events were detected across all samples (**Supplementary Table 2**), occurring in a frequency order of SE> A3SS> A5SS> MXE> RI (**Figure 2**). Applying an FDR threshold of padj < 0.05 identified 274 statistically significant differential splicing events across 230 discrete genes (**Supplementary Table 3**). Consistent with the global distribution, SE was the predominant splicing category, accounting for 197 events, followed by A3SS with (25 events), RI with (20 events), MXE with (20 events), and A5SS with (12 events). These findings indicate that exon skipping represents the primary post-transcriptional mechanisms regulated by PRELP in BUF1088 melanoma cells.

**Figure 2:**
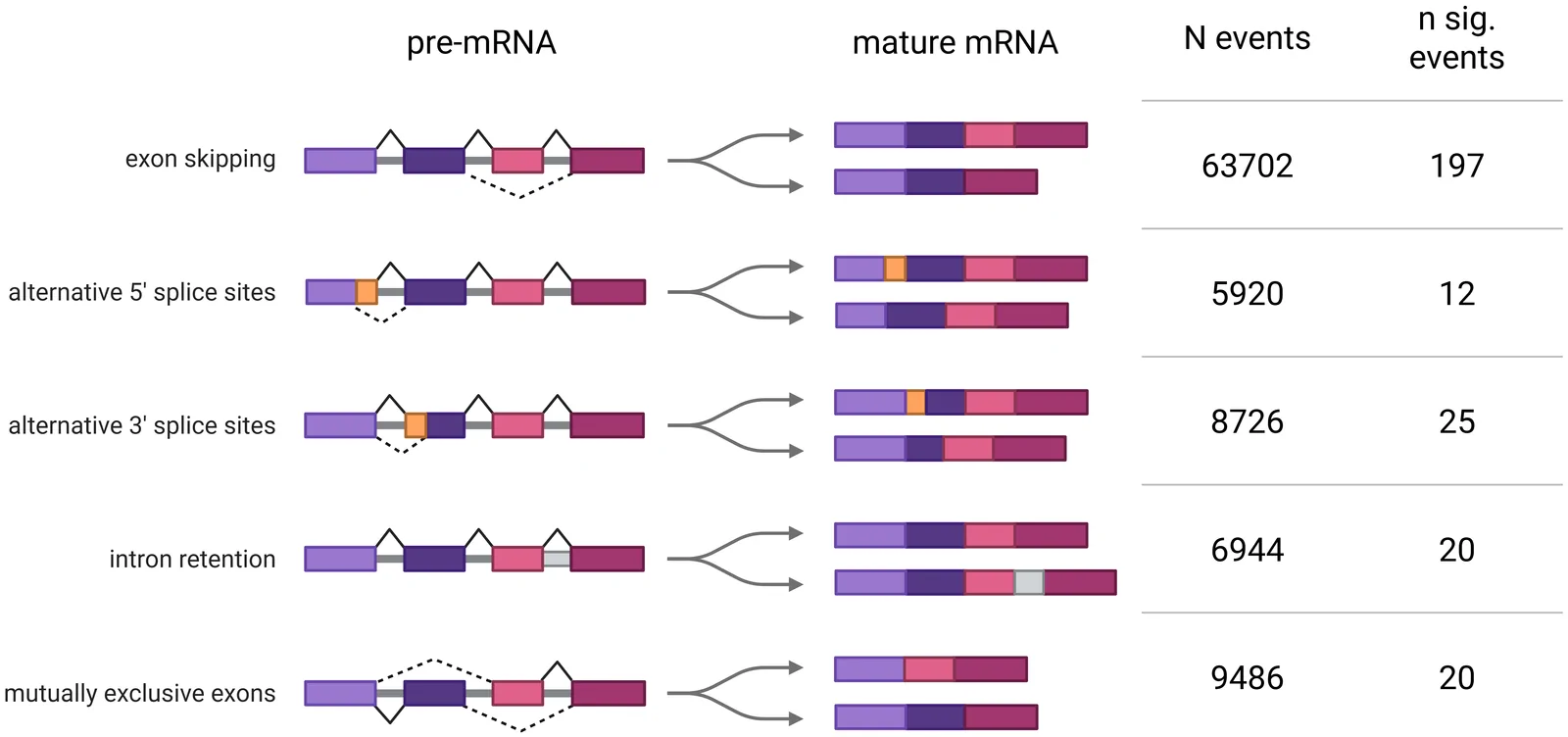
Classification and distribution of AS events modulated by PRELP. Schematic diagrams illustrating the five major classes of alternative splicing (AS) analyzed in the RNA-seq dataset: skipped exon (SE), alternative 5′ splice site (A5SS), alternative 3′ splice site (A3SS), retained intron (RI), and mutually exclusive exon (MXE). Quantitative summary table shows the total number of detected AS events and the number of statistically significant differential splicing events identified in PRELP-high compared with PRELP-low BUF1088 melanoma cells. Statistical significance was defined using a false discovery rate threshold of adjusted p value < 0.05. Figure created with BioRender.

The top 15 most significant alternatively spliced genes are summarized in **Table 2A**. Among these, AAR2, SACM1L, CD22, and ACTB inhibited the strongest statistical significance. SE was validated as the predominant modality among these top-ranked genes, including AAR2, MYO5A, WARS1, CRACD, BTN3A3, TNS1, ARL1, VWDE, IL24, CMC2, and HNRNPA3. Additionally, alternative 3′ splice site events were detected in SACM1L and CD22, while CCDC7 showed an alternative 5′ splice site event and ACTB displayed intron retention. Overall, these results indicate that altered PRELP expression status is associated with specific changes in the transcript processing.

**Table 2:** Top differentially alternatively spliced genes and corresponding expression dynamics.

A: Top 15 genes with the most significant differential alternative splicing events between PRELP-high and PRELP-low BUF1088 melanoma cells, Candidates are ranked by raw p value and false discovery rate (FDR).			
Gene symbol	PValue	FDR	Splicing type
AAR2	0	0	SE
SACM1L	0	0	A3SS
CD22	0	0	A3SS
ACTB	0	0	RI
CCDC7	8.88E-16	5.26E-12	A5SS
MYO5A	7.77E-16	1.65E-11	SE
WARS1	4.11E-14	3.27E-10	SE
CRACD	3.91E-14	3.27E-10	SE
BTN3A3	3.45E-14	3.27E-10	SE
TNS1	2.50E-14	3.27E-10	SE
ARL1	3.53E-14	3.27E-10	SE
VWDE	4.63E-14	3.28E-10	SE
IL24	6.13E-12	3.00E-08	SE
CMC2	7.01E-12	3.19E-08	SE
HNRNPA3	8.93E-12	3.79E-08	SE

B: Corresponding differential expression parameters for these top spliced targets shown as log2 fold change (PRELP-high vs PRELP-low) and Benjamini-Hochberg adjusted p values derived from differential gene expression analysis.			
Gene_name	Log2 fold change		Padj
AAR2	-0.132		0.528
SACM1L	0.074		0.716
CD22	0.557		0.003
ACTB	-0.17		0.167
CCDC7	-0.057		0.962
MYO5A	-0.43		0
WARS1	1.103		0
CRACD	0.158		0.695
BTN3A3	0.848		0
TNS1	-0.295		0.612
ARL1	-0.048		0.837
VWDE	0.497		0.259
IL24	3.417		0
CMC2	0.023		0.929
HNRNPA3	-0.235		0.052

### Integration of transcript structure and abundance

To determine whether genes undergoing significant AS also showed changes in overall transcript abundance, the top differentially alternatively spliced genes were compared with differential gene expression results (**Table 2B**). This analysis identified a subset of highly prioritized genes that are concurrently regulated at both the structural and transcriptional levels, including CD22, MYO5A, WARS1, BTN3A3, and IL24. Both CD22 and IL24 showed negative ΔPSI values (−0.272), consistent with reduced exon inclusion in PRELP-high cells. Similarly, BTN3A3 contained two independent exon exclusion events (ΔPSI = −0.213 and −0.718), whereas MYO5A showed an increased exon inclusion (ΔPSI 0.313). Notably, WARS1 exhibited multiple significant splicing events with five distinct splicing junctions shifting toward positive inclusion (ΔPSI values: 0.408, 0.243, 0.213, 0.370, and 0.211). Parallel expression analysis revealed that IL24 was the most highly upregulated gene in PRELP-high cells (log2 FC = 3.417), while WARS1 and BTN3A3 expression levels were also upregulated. In contrast, MYO5A expression was significantly downregulated in PRELP-high cells.

Sashimi plot analysis confirmed this differential IL24 isoform usage between PRELP-high and PRELP-low BUF1088 melanoma cells capturing a clear shift in exon-junction architecture for IL24 transcripts across replicates, with inclusion levels ranging from 0.65 to 0.84 and an IncLevelDifference/ΔPSI of −0.272, consistent with altered exon usage. Sashimi plots for additional differentially spliced genes are shown in **Supplementary Figure 1**.

Replicate consistency across these targets was further verified by hierarchical clustering (**Figure 3A**) with WARS1, IL24, CD22, and BTN3A3 enriched in PRELP-high cells and MYO5A and HNRNPA3 reduced. Notably, several top-ranked splicing targets, including AAR2, SACM1L, ACTB, CCDC7, and TNS1, showed no significant changes in overall transcript abundance. These findings suggest that PRELP-associated transcriptomic remodeling involves both splicing-dependent regulation and coordinated changes in gene expression.

**Figure 3:**
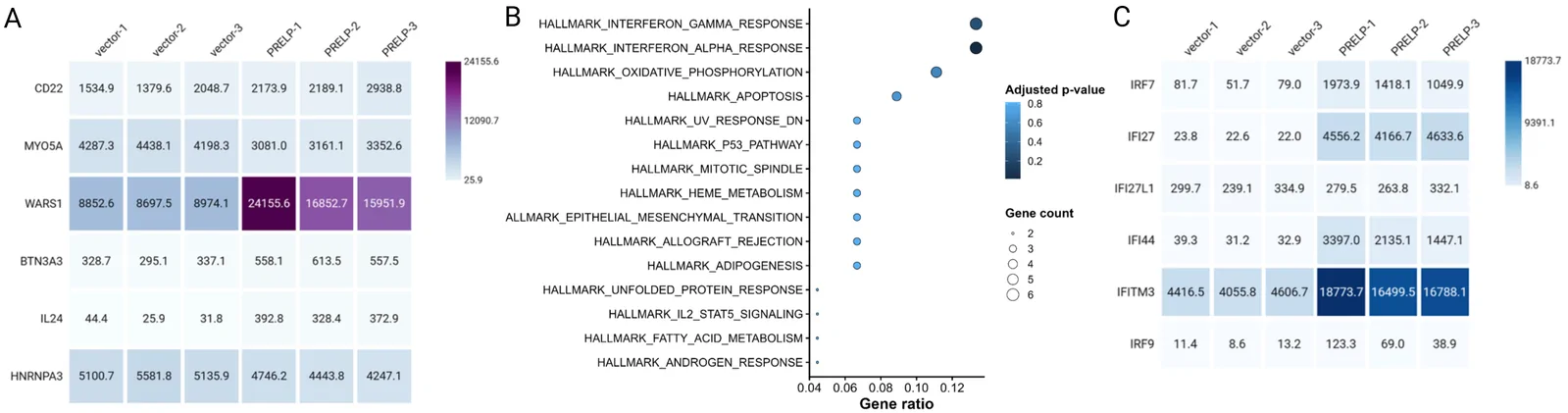
PRELP-mediated induction of genome-wide alternative splicing enriched in IFN-gamma response pathways. **(A)** Unsupervised hierarchical clustering heatmap showing normalized expression levels (z scores) of significantly differentially alternatively spliced genes across PRELP-low vector-control (n=3) and PRELP-high (n=3) BUF1088 melanoma samples. Rows denote individual spliced genes and columns represent biological replicates; color intensity corresponds to relative gene expression (red, high expression; blue, low expression). **(B)** Hallmark pathway enrichment analysis genes displaying significant differential AS between PRELP-high and PRELP-low melanoma cells. In the bubble plot the x-axis represents the gene ratio (number of spliced genes divided by total genes assigned to the pathway. Bubble size is proportional to the overall gene count, and bubble color indicates the adjusted p value calculated via hyper-geometric testing with Benjamin-Hochberg correction. Pathways with larger bubbles and lower adjusted p values represent stronger enrichment trends. **(C)** Heatmap showing normalized log2-transformed expression profiles of key genes comprising the hallmark IFN-gamma response gene set across PRELP-high and PRELP-low melanoma cell replicates.

### Link of PRELP-associated alternative splicing to interferon signaling

To understand the functional impact of these structural changes, pathway enrichment analysis of the 230 significantly alternatively spliced genes was performed and identified strong associations with immune response pathways, particularly the hallmark IFN-gamma and IFN-alpha response signatures (**Figure 3B**). Additional enriched pathways included oxidative phosphorylation, apoptosis, UV response, p53 pathway, mitotic spindle, heme metabolism, epithelial–mesenchymal transition, and allograft rejection indicating broad impact of PRELP-associated transcriptomic remodeling beyond isolated AS events involving coordinated regulation of immune signaling, metabolic reprogramming, and apoptosis-related pathways.

Consistent with the enrichment of IFN pathways, PRELP-high melanoma cells compared with PRELP-low vector-control cells demonstrated a coordinated upregulation of downstream IFN-stimulated genes (**Figure 3C**). In particular, IFI27, IFI44, IRF7, and IFITM3 showed higher expression across PRELP-high biological replicates with IFITM3 displaying the highest total abundance. In contrast, IFI27L1 showed relatively comparable expression between groups, while IRF9 demonstrated only modest expression changes indicating a selective rather than generalized activation of IFN signaling.

### Identification of alternative splicing events and validation of transcript expression

To validate the RNA-seq and alternative splicing predictions, we examined the expression of selected IFN-response pathway genes, including IRF7, IFI27, IFI44, and IFITM3, as well as differentially expressed splicing-associated genes, including WARS1 and IL24, across both the PRELP-high and PRELP-low primary discovery BUF1088 cell line and BUF1102 melanoma cells as independent cell line.

RT-qPCR analysis showed significant differences in the mRNA expression levels of IRF7, IFI27, IFI44, IFITM3, WARS1, and IL24 between PRELP-high and PRELP-low melanoma cell lines, BUF1088 (**Figure 4A**) and BUF1102 (**Supplementary Figure 2A**). These findings were consistent with the RNA-seq data and support activation of an IFN-response-associated transcriptional program in PRELP-high melanoma cells.

**Figure 4:**
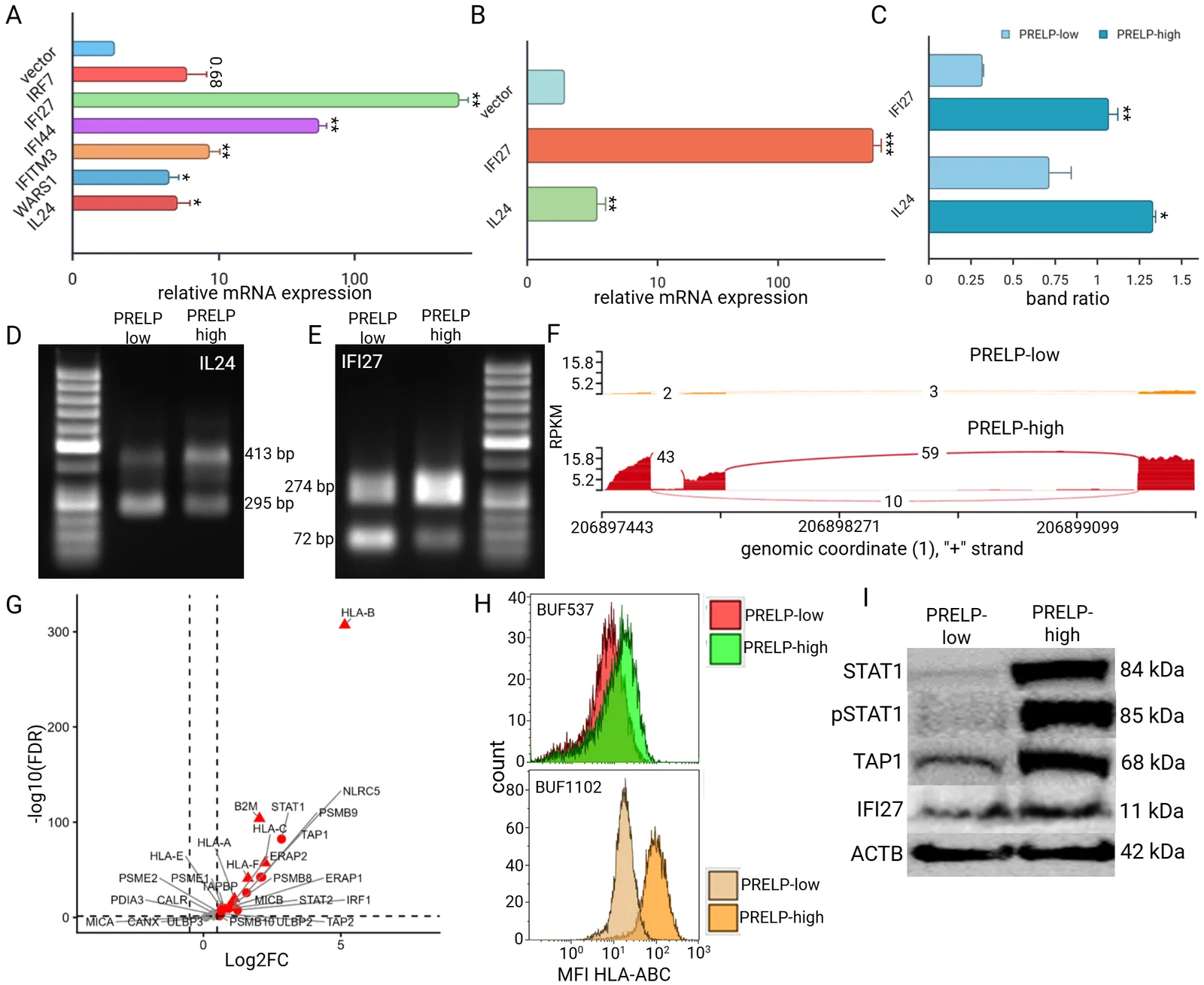
Experimental validation of PRELP-associated IFN-response, exon-skipping, and MHC-I antigen-presentation changes. **(A)** Relative mRNA expression of representative IFN-associated genes (IRF7, IFI27, IFI44, IFITM3, WARS1, and IL24) measured by RT-qPCR in PRELP-overexpressing BUF1088 melanoma cells relative to vector-control cells (log10 scale; n=3). **(B)** Isoform-specific RT-qPCR quantification of exon-skipped transcript variants for IFI27 and IL24 using custom splice-variant-specific primers in PRELP-overexpressing versus control cells. **(C, D)** Densitometric quantification **(C)** and representative agarose gel electrophoresis **(D)** of RT-PCR amplification products evaluating exon inclusion versus skipping ratios for IL24 in PRELP-low and PRELP-high cells. **(E)** Representative agarose-gel electrophoresis of IFI27 RT-PCR products validating differential splice variant abundance. **(F)** RNA-seq Sashimi plots illustrating differential junction coverage at the IL24 locus in three biological replicates per condition. Numbers on curved arcs quantify raw read coverage supporting specific junction events. Lower genomic track depicts reference exon–intron structure. **(G)** Volcano plots depict the differential gene expression of MHC-I APM components comparing PRELP-overexpressing cells with vector control cells. The x-axis represents log_2_FC, while the y-axis shows −log_10_(FDR). Red symbols mark significantly upregulated genes, while grey symbols indicate non-significant changes. **(H)** Representative flow-cytometric histogram of surface HLA-ABC expression in vector-control and PRELP-overexpressing melanoma cells. **(I)** Western blot analysis of total STAT1, pSTAT1, TAP1, and IFI27 protein levels in vector-control and PRELP-overexpressing cells. ACTB served as the loading control. * P<0.05, ** P<0.01, *** P<0.001.

Gel-based RT-PCR validated selected exon-skipping events predicted by rMATS for IFI27 and IL24 in BUF1088 (**Figure 4B**) and BUF1102 (**Supplementary Figure 2B**). Both targets resolved into two transcript isoforms, corresponding the long (exon-included) and short (exon-skipped) transcript variants (**Figure 4C**). In line with the sequencing data, the longer transcript isoform for both IFI27 (**Figure 4D**) and IL24 (**Figure 4E**) was reduced in PRELP-high melanoma cells compared with PRELP-low cells further supporting PRELP-associated regulation of IL24 transcript processing (**Figure 4F**).

Together, these results indicate that PRELP overexpression is associated not only with increased expression of IFN-response genes, including IFI27 and IL24, but also with altered isoform usage of selected transcripts.

### Enhancement of antigen presentation and core JAK/STAT1 signaling

Because IFN networks are central regulators of tumor immunogenicity, we investigated whether PRELP-driven transcriptomic changes altered the antigen presentation machinery (APM) of melanoma cells. Transcriptomic profiling of PRELP-overexpressing melanoma cells revealed a pronounced and coordinated induction of multiple MHC class I APM components. Volcano plot analysis demonstrated a robust upregulation of classical MHC-I heavy chains (HLA-A, HLA-B, HLA-C), the invariant light chain B2M, peptide transporters (TAP1, TAP2), immunoproteasome subunits (PSMB8, PSMB9), and the endoplasmic reticulum aminopeptidases ERAP1 and ERAP2 (**Figure 4G**).

To determine whether these transcriptional changes translated into altered cell surface antigen presentation, PRELP-overexpressing melanoma cells displayed consistently elevated surface expression of MHC-I (**Figure 4H**) compared to their respective vector controls indicating enhanced antigen presentation capacity.

To further characterize the upstream signaling events driving these changes, Western blot analyses for JAK/STAT pathway components were performed in Buf1088 cells. PRELP overexpression increased total STAT1 expression, enhanced STAT1 phosphorylation at Tyr701 (pSTAT1) and elevated IFI27 and TAP1 protein abundance (**Figure 4I**). These results indicate that PRELP expression promotes antigen presentation capacity via activation of the canonical IFN/STAT1 axis.

### Predictive utility of PRELP-related gene signatures in immunotherapy cohorts

Given the enrichment of IFN-response pathway and antigen presentation in PRELP-high melanoma cells, we investigated whether PRELP-related genes, including PRELP, IRF7, IFI27, IFI27L1, IFI44, IFITM3, WARS, and IL24, were linked to immunotherapy response in melanoma patients across three clinical melanoma datasets using the TIGER portal. In the GSE115821 cohort (anti-CTLA-4, anti-PD-1 and anti-CTLA-4+anti-PD-1), baseline PRELP expression was significantly higher in clinical responders than in non-responders (Wilcoxon p = 0.038). IRF7 and IFI44 showed a trend toward increased expression in responders that did not reach statistical significance (both p = 0.053), whereas IFI27L1 expression was significantly lower in responders (p = 0.0095). No significant baseline differences were observed for IFI27, IFITM3, WARS, and IL24 (p = 0.72, 0.68, 0.98, and 0.72, respectively) in this cohort (**Figure 5**).

**Figure 5:**
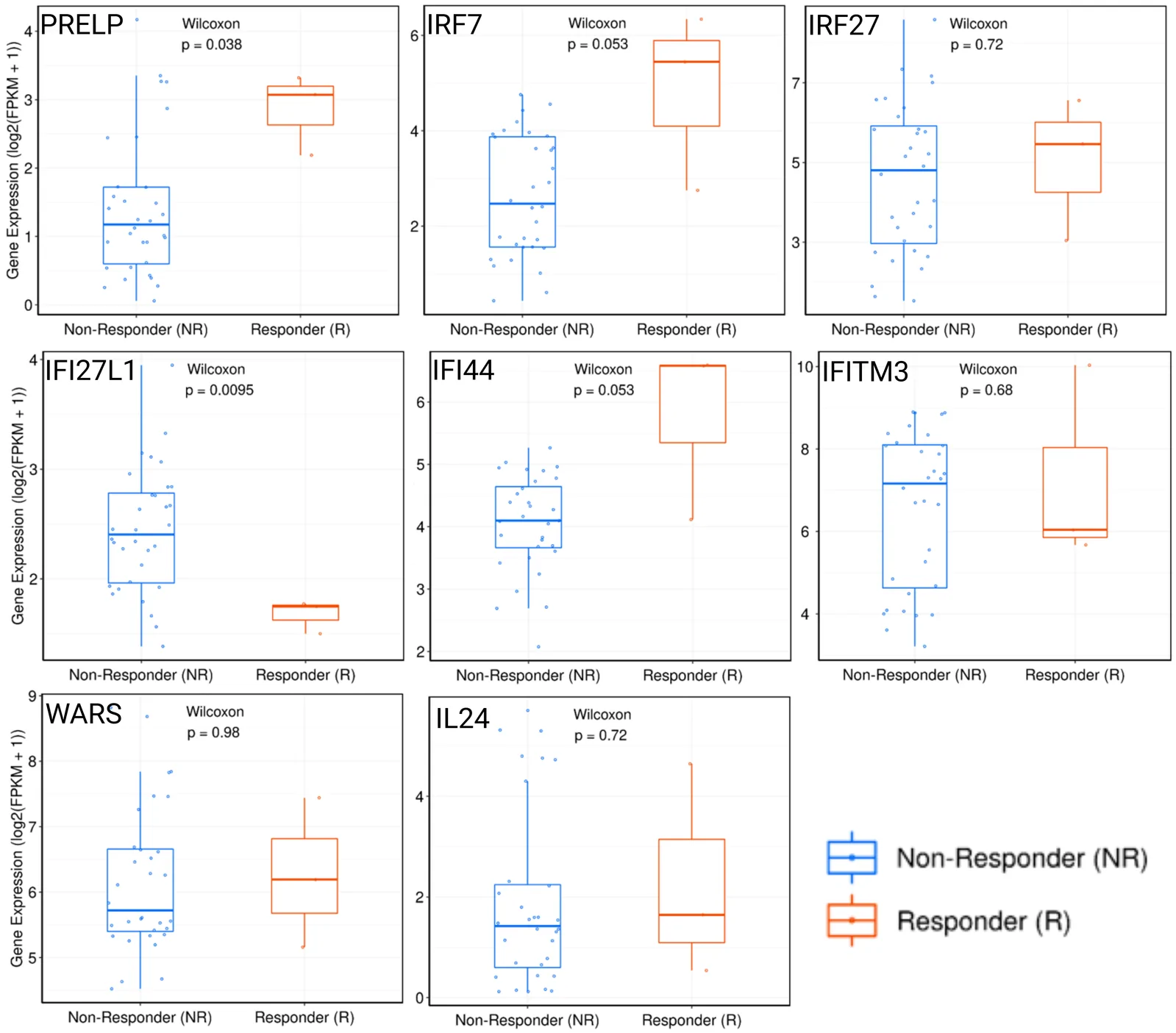
Correlation of high PRELP-related gene signatures with therapy response in melanoma patients. Boxplots showing expression levels of individual genes, measured as log2(FPKM + 1), in in pre-treatment baseline biopsies from melanoma patients in the GSE115821 cohort (n=37). Patient samples were stratified by clinical outcome following therapy: responders (n=3) and non-responder (n=34). Center line, median; box limits, upper and lower quartiles; whiskers, 1.5× interquartile range; points, individual patient values. Statistical significance between groups was computed using two-tailed Wilcoxon rank-sum test.

To evaluate the collective biomarker potential of these targets, a composite signature consisting of PRELP, IRF7, IFI27, IFI27L1, IFI44, IFITM3, WARS, and IL24 was constructed and evaluated across two independent melanoma ICB cohorts: an anti-CTLA-4 cohort (24) and the PRJEB23709 cohort, which included patients treated with anti-PD-1 monotherapy or combined anti-PD-1/anti-CTLA-4 therapy (25) (**Figure 6**). In both the anti-CTLA-4-treated melanoma cohort and the PRJEB23709 ICB cohort, responders exhibited significantly higher signature scores than non-responders, as determined by the Wilcoxon rank-sum test with p = 0.027 and p = 0.00021, respectively. These findings suggest that the PRELP-related IFN response programs are linked to favorable clinical responses across multiple modalities of immune checkpoint blockade (ICB) in melanoma.

**Figure 6:**
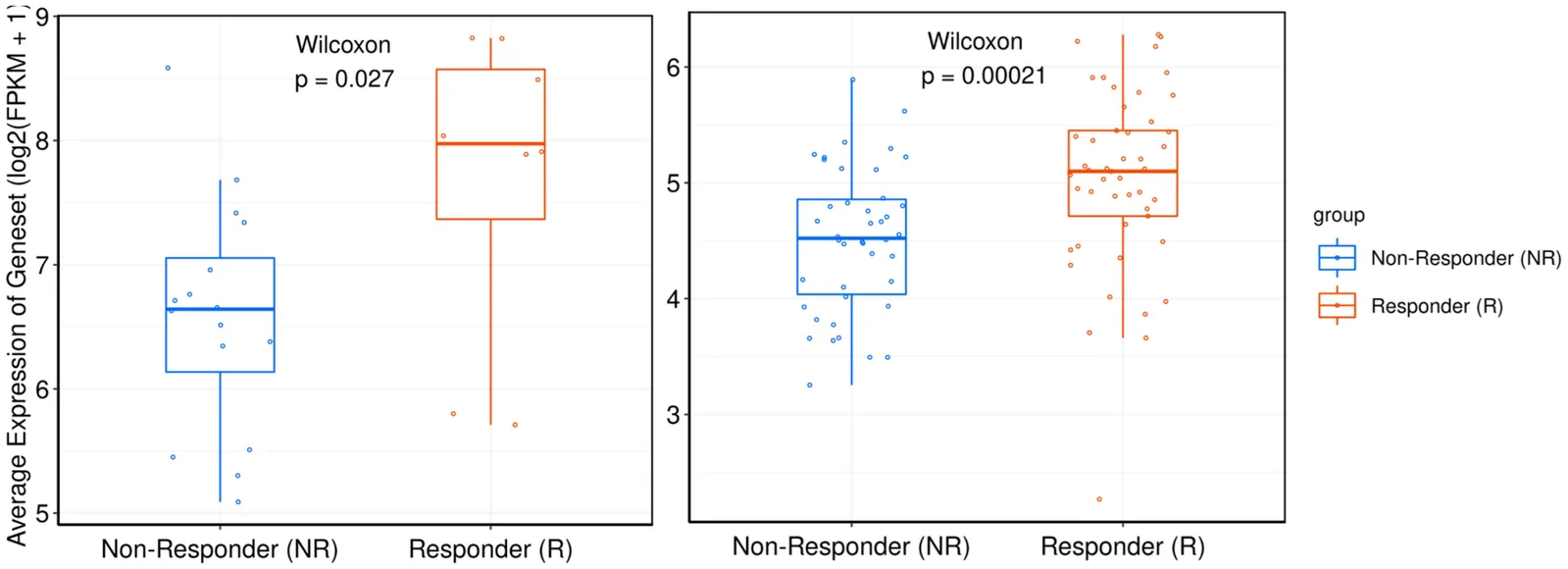
Validation of PRELP-related gene signatures in melanoma patients receiving ICB. Comparative evaluation of average expression of the PRELP-related gene signatures, measured as log2(FPKM + 1) between non-responders (NR) and responders (R) across clinical ICB datasets accessed via the TIGER database. A: Anti-CTLA-4 treatment cohort (24): NR, n = 16; R, n = 8. B: PRJEB23709 cohort treated with anti-PD-1 monotherapy or combination of anti-CTLA-4 plus anti-PD-1 therapy (4): NR, n = 42; R, n = 49. Box plots show median, interquartile range, and range. Statistical differences were evaluated using two-tailed Wilcoxon rank-sum test.

### Single-cell mapping of PRELP and PRELP-related gene signatures in the melanoma tumor microenvironment

To elucidate the cellular architecture driving these transcriptional signatures, we performed scRNA-seq analysis using the GSE115978 melanoma dataset (7, 186 high-quality single-cell profiles from 33 melanoma tumors derived from 31 patients) (6). This analysis resolved the primary cellular components of the melanoma TME distinguishing CAFs, endothelial cells, macrophages, B cells, CD4+ T cells, CD8+ T cells, NK cells, and malignant cells (**Figure 7A**). CAFs and endothelial cells formed distinct stromal clusters, while immune and malignant populations occupied separate coordinates in the UMAP space.

**Figure 7:**
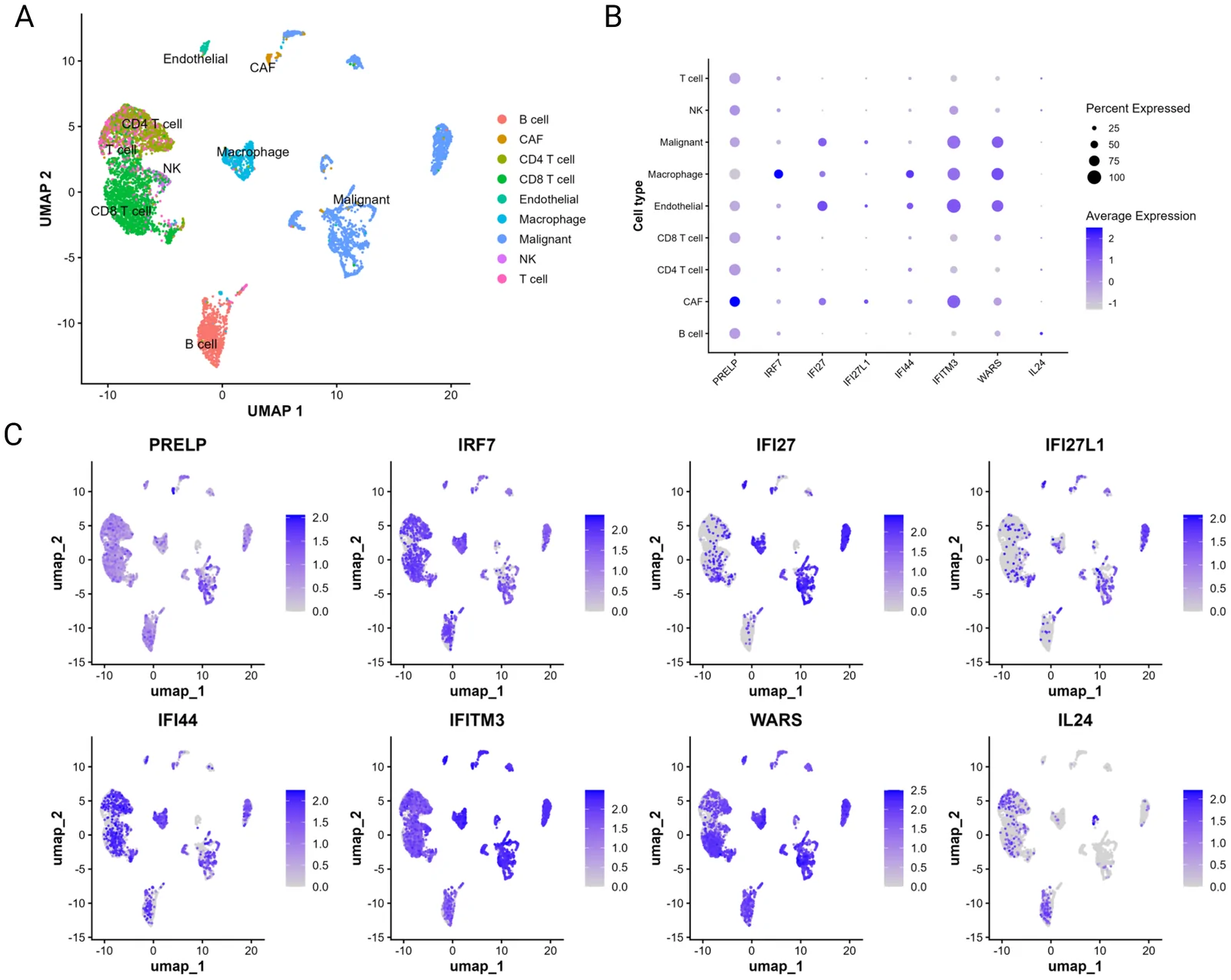
Single-cell transcriptomic resolution of PRELP-related gene signatures across the melanoma microenvironment. **(A)** UMAP visualization embedding of single-cell transcriptomes from the GSE115978 melanoma dataset (6) shows major microenvironmental cell populations, including tumor cells, CAFs, endothelial cells, macrophages, B cells, CD4+ T cells, CD8+ T cells, NK cells, and other T cells. Points represent individual cells colored by broad cell lineage annotation. **(B)** The accompanying dot plot summarizes the expression levels and cellular prevalence of PRELP and PRELP-related gene signatures across annotated cell types. Dot size indicates the percentage of cells expressing each gene, and color gradient represents scaled average expression. **(C)** UMAP plots of PRELP and PRELP-related gene signatures in GSE115978 melanoma Single cell feature density plots were projecting log-normalized expression of PRELP, IRF7, IFI27, IFI27L1, IFI44, IFITM3, WARS/WARS1, and IL24 onto the UMAP embedding of annotated melanoma single cells.

Characterization of individual gene expressions revealed that PRELP expression was strongly enriched within the CAF compartment with limited baseline expression in either malignant and immune cells (**Figure 7B**). In contrast, the broader PRELP-related gene signatures displayed widespread and more heterogeneous expression patterns across multiple cell populations. For instance, IRF7 expression was most prominent localized in macrophages, wherea*s* IFI27, IFI44, IFITM3, *and* WARS/WARS1 were detected across malignant, stromal, and immune compartments (**Figure 7C**). These single cell insights indicate that PRELP is primarily a stromal/CAF-associated marker, whereas IFN-related transcriptional programs operate as a distributed network involving multiple cell types within the melanoma microenvironment.

### Identification of PRELP-enriched fibroblast states by CAF subclustering

To achieve a more granular understanding of PRELP-positive stromal dynamics, CAF-focused subclustering was performed using CAF-curated signatures. This resolved four primary functional CAF states including ECM myCAFs, IFN-responsive CAFs (IFN-CAFs), inflammatory CAFs (i-CAFs), and antigen-presenting CAFs (apCAFs) (**Figure 8A**). UMAP visualization demonstrated distinct heterogeneity within the CAF compartment with ECM myCAFs representing the dominant fibroblast state and occupying a transcriptionally distinct region of the UMAP space. Inflammatory, antigen-presenting, and IFN-responsive CAFs were positioned cluster in close proximity suggesting overlapping, yet distinct CAF activation programs (**Figure 8B**). Expression mapping across these sub-clusters revealed clear, state-specific patterns for PRELP and its related interferon network. PRELP expression was most prominent in ECM myCAFs and IFN-responsive CAFs with lower expression in inflammatory CAFs (**Figure 8B**). Among IFN-related genes, IRF7 and IFI44 were heavily restricted to the IFN-responsive CAF subtype, supporting their activation state. IFI27 and WARS were enriched in ECM myCAFs, while IFITM3 demonstrated broader distribution across multiple CAF identities. IFI27L1 and IL24 displayed comparatively limited expression.

**Figure 8:**
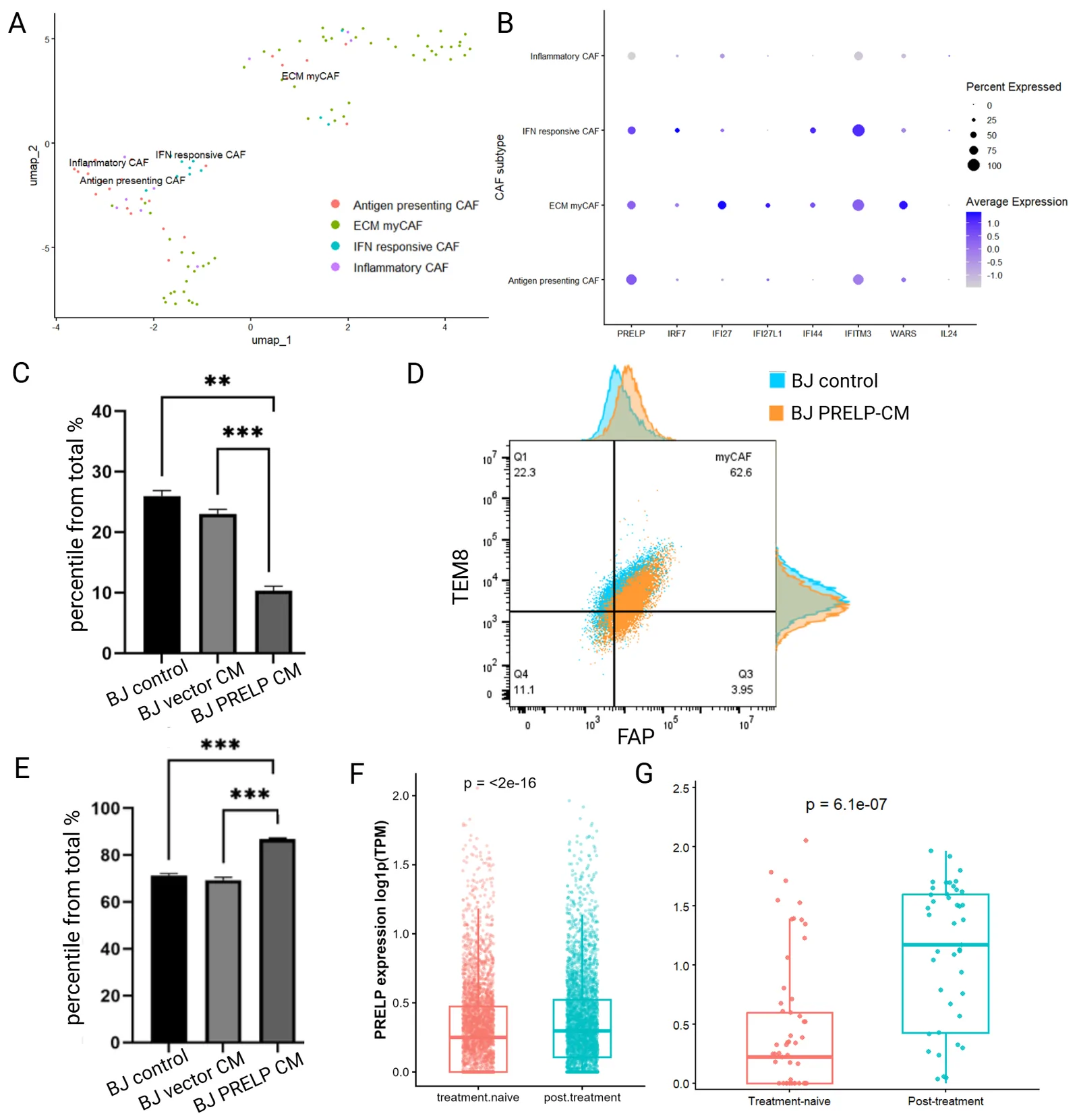
PRELP drives stromal reprogramming toward an ECM-rich and IFN-responsive CAF phenotype. **(A)** High resolution UMAP sub-clustering of CAFs from the GSE115978 melanoma scRNA-seq dataset identifies four CAF subtype states: ECM myCAF, IFN-responsive CAF, inflammatory CAF, and antigen-presenting CAF. Each point represents a CAF and is colored according to subtype annotation. **(B)** The dot plot summarizes expression of PRELP and IFN-related genes across the four CAF subtypes. Dot size indicates the percentage of cells expressing each gene, while color intensity represents scaled average expression. PRELP is enriched primarily in ECM myCAF and IFN-responsive CAF populations, whereas IFN-associated genes, including IRF7, IFI44, IFITM3, and WARS, show subtype-specific expression, with strongest interferon-related signal in IFN-responsive CAFs. **(C)** Quantitative flow cytometric analysis of FAP-positive fibroblasts among total BJ cells cultured under baseline control conditions or exposed to conditioned medium (CM) from vector-control or PRELP-overexpressing melanoma cells (n=3). **(D)** Representative flow cytometric bivariate density plots quantifying the proportion of FAP+ and TEM8+ myCAFs within BJ fibroblasts following CM exposure. **(E)** Relative abundance of IFNαβ-myCAFs calculated as fraction of the total remaining myCAF compartment following exposure to PRELP-overexpressing melanoma cell CM (n=3). **(F, G)** Boxplots showing PRELP expression levels stratified by treatment status across all annotated microenvironmental cell types **(F)** and specifically within the CAF compartment **(G)** in the GSE115978 cohort. Statistical significance assessed via two-tailed Student’s t-test or Wilcoxon rank-sum test (** P<0.01, *** P<0.001).

To determine whether melanoma-derived PRELP could functionally influence these CAF states, fibroblasts were exposed to conditioned medium derived from PRELP-overexpressing BUF1088 melanoma cells. Exposure to PRELP-high conditioned medium reduced the proportion of conventional FAP-positive myCAFs and altered the distribution of the remaining subpopulations (**Figure 8C**). This shift was driven by a distinct downregulation of FAP expression rather than direct cell death or depletion of the fibroblast pool (**Figure 8D**). Within the residual myCAF compartment IFNαβ-myCAFs were substantially expanded (**Figure 8E**). Consistent with this phenotypic redistribution, qPCR analysis validated that fibroblasts exposed to PRELP-high conditioned medium revealed a significant upregulation of IFN-associated genes IFI27 (**Supplementary Figure 3A**), IRF7 (**Supplementary Figure 3B**), IFITM3 (**Supplementary Figure 3C**), and WARS1 (**Supplementary Figure 3D**), whereas IL24 (**Supplementary Figure 3E**) expression remained static. This experimentally observed redistribution was consistent with the single-cell association of PRELP with both ECM-related and IFN-responsive fibroblast states. These data indicate that melanoma-derived PRELP can actively remodel fibroblast phenotypes driving the transition of stromal fibroblasts from an ECM-dominant phenotype toward an IFN-responsive state.

### Treatment-associated increase of PRELP expression within the stroma

Further analysis of the GSE115978 dataset showed that PRELP expression was significantly elevated in post-treatment samples compared with treatment-naive samples within the GSE115978 cohort (p 2 × 10^-16^, **Figure 8F**; **Supplementary Figure 4**). This increase was particularly pronounced within the CAF compartment, where post-treatment CAFs displayed a drastic increase in PRELP transcript abundance relative to baseline CAFs (p 6.1 × 10^-7^, **Figure 8G**).

Together with the conditioned-medium experiments, these clinical findings imply that treatment-associated PRELP induction serves as an active driver stroma remodeling. Increased PRELP after treatment may promote suppression of classic immune-evasive ECM-myCAF features, preferentially favor the enrichment of IFN-responsive fibroblast states that may alter therapeutic vulnerability.

### Independent validation of PRELP stromal localization in the GSE72056 cohort

To independently assess the reproducible cellular distribution of PRELP in a distinct melanoma cohort, the metastatic melanoma scRNA-seq dataset GSE72056 was analyzed. UMAP visualization identified the major cellular constituent of the metastatic melanoma TME, including malignant cells, T cells, B cells, macrophages, endothelial cells, CAFs, and NK cells (**Supplementary Figure 5A**). Because the original metadata did not distinguish between CD4+ and CD8+ T cell subsets, all T cells were represented as a single annotated population.

Consistent with primary observations, PRELP expression was predominantly localized to stromal regions corresponding to CAF-enriched stromal populations (**Supplementary Figure 5B**). This independent cohort confirms that PRELP acts as a highly reproducible, stromal-confined marker rather than a generalized or ubiquitous IFN-responsive transcript. In contrast to PRELP’s strict stromal compartmentalization, its IFN-related signature genes (IRF7, IFI27, IFI44, IFITM3, and WARS/WARS1) displayed broader expression patterns across multiple cellular compartments. including immune, malignant, and stromal cell populations, whereas IL24 exhibited a comparatively more restricted distribution (**Supplementary Figure 6**).

Taken together, these dual-cohort cross-validations confirm that PRELP serves as a specific stromal anchor reflecting a highly integrated, multilineage IFN-responsive TME.

## Discussion

The present study establishes PRELP as a central component of a broader, coordinated, immune-supportive transcriptional state in melanoma characterized by extensive splicing remodeling, activation of IFN-responsive transcriptional programs, improved antitumor immunity and enhanced responsiveness to ICB. Transcriptional profiling of PRELP-high BUF1088 melanoma cells showed widespread posttranscriptional remodeling affecting 230 genes with predominance of exon-skipping events. In parallel, pathway enrichment analyses confirmed robust activation of both IFN-gamma and IFN-alpha response pathways, marked by upregulation of downstream effectors such as IFI27, IFI44, IRF7, and IFITM3.

The resulting composite PRELP-related signature was consistently enriched in clinical responders across multiple immunotherapy cohorts pointing to a state, where preserved IFN responsiveness and extensive transcriptomic remodelling intersect to favor antitumor immunity.

These observations extend our previous work, showing that PRELP loss in melanoma correlates with reduced expression of MHC class I APM components, diminished IFN-γ pathway signaling, lower intratumoral T cell density, and reduced CCL5, whereas PRELP restoration in melanoma cell lines enhanced MHC class I and CCL5 expression, while suppressing malignant phenotypes (15). The present findings broaden this model by showing that PRELP-associated states involve not only widespread changes in overall gene expression, but also substantial remodeling of transcript isoforms. Since many significantly alternatively spliced genes under PRELP influence did not exhibit significant changes in total transcript abundance, PRELP-associated environmental control appears to operate across distinct, independent layers of gene expression. These results align with accumulating evidence that alternative splicing events contributes to tumor plasticity, immune regulation, and therapeutic response in melanoma (16–19).

Among the pathways associated with PRELP-high melanoma cells, IFN signaling emerged as a central pillar of the PRELP-high phenotype. IFN pathways play a critical role in antitumor immunity due to their capacity to boost antigen presentation, recruit immune cells, and facilitate tumor cell recognition by cytotoxic lymphocytes. Because melanoma frequently downregulates IFN responsiveness to evade immune elimination (27), preserving this capacity is biological critical. Indeed, defects in downstream components, such as JAK1, JAK2, and B2M, are key drivers of acquired resistance to ICB in melanoma (3). At the same time, IFN signaling is highly context-dependent and can exert both antitumor and protumor effects depending on the microenvironmental cues and the integrity of downstream immune pathways (28, 29). Therefore, our findings should not be interpreted as a simplistic claim that increased IFN activity alone predicts favorable outcome. Instead, PRELP expression likely identifies a highly organized, stable biological program that maintains functional IFN responsiveness supported by Western blot confirmation of increased STAT1 expression and phosphorylation alongside immune-supportive transcriptional configurations (4, 25).

Our single-cell analyses provide important spatial and cellular context for these interactions. Across two independent melanoma scRNA-seq datasets (GSE115978 and GSE72056), PRELP expression was consistently enriched within CAF populations and showed comparatively limited expression in malignant and immune cells. In contrast, IFN-related genes, such as IRF7, IFI27, IFI44, IFITM3, and WARS1, displayed broader expression patterns across immune, malignant, endothelial, and stromal compartments. This contrast proves that PRELP is not merely a redundant marker of general IFN exposure, but rather a dedicated stromal signal associated with an IFN-responsive TME. Further characterization of the CAF compartment revealed that PRELP expression marks specific ECM-myCAF-like and IFN-responsive fibroblast states. Interestingly, PRELP^+^ CAFs featuring similar ECM-remodelling and immunoregulatory profiles were recently identified in colorectal cancer liver metastases (30). In our study, the observed reduction in traditional ECM-myCAFs following exposure to PRELP-high conditioned medium may have important functional consequences. Because this specific myCAF subset has been shown to drive tumor-cell PD-L1 expression, thereby fostering an immunosuppressive microenvironment and facilitating immune escape (31), its targeted depletion or transition may disrupt stromal support for PD-L1-mediated immune evasion and improve susceptibility of melanoma cells to T cell-mediated immune destruction.

Given the recognized functional heterogeneity of CAFs, these finding suggest that PRELP-positive fibroblasts may occupy a specialized niche that integrates ECM structural integrity with local immunoregulation (9, 10, 32). Consistent with this interpretation, PRELP expression was significantly increased in post-treatment samples, particularly within the CAF compartment. Together with emerging evidence that distinct fibroblast populations can either promote immune exclusion or support antitumor immunity, these findings raise the possibility that PRELP-positive fibroblasts contribute to the establishment of an immune-supportive stromal microenvironment (9, 10, 32).

From a translational perspective, our clinical cohort analyses demonstrate that PRELP is unlikely to function as a standalone predictive biomarker. Given the multi-faceted nature of ICB resistance, single-gene predictors rarely capture the collaborative dynamics of tumor-intrinsic pathways, IFN competence, stromal architecture, and immune cell density (1, 4, 24, 25). Instead, the data support its integration into a broader composite transcriptional signature incorporating IFN response genes, ECM-associated pathways and transcript isoforms. A PRELP-centred signature integrating ECM-associated genes, IFN effectors (e.g., STAT1, TAP1, IRF7), and isoform-level splice features provides a more biologically robust and clinically portable predictor of therapeutic response.

Future studies should focus on both mechanistic and clinical validation. Mechanistically, functional PRELP perturbation should be performed in both melanoma cells and fibroblasts, ideally using co-culture models or patient-derived organoid systems to delineate ECM-dependent signaling axes and multi-lineages immune crosstalk during PRELP disruption. Integrating long-read RNA sequencing with targeted splice variant validation would be an essential step to establish whether specific isoforms alter coding potential or generate targetable neoepitopes. Furthermore, employing spatial transcriptomics or multiplexed tissue imaging will clarify whether PRELP-positive fibroblasts preferentially colocalize with T cell-permissive versus T cell-excluded niches. Functionally, rigorous antigen-presentation assays, T cell killing assays, and IFN perturbation experiments will be needed to definitively classify whether PRELP acts as a direct upstream driver of immune sensitivity, a downstream consequence of local inflammation or an interactive coordinator of both processes. Translationally, the most promising path forward is the prospective validation of a pre-treatment, PRELP-centered composite biomarker signature within the longitudinal melanoma biopsy cohorts that integrates ECM state, IFN responsiveness, and isoform-level remodeling in pretreatment melanoma biopsy cohorts to evaluate its clinical utility as a predictor of long-term immunotherapy response.

In summary, the present study highlights an integrated model, in which PRELP coordinates a distinct melanoma microenvironment characterized by ECM remodeling, stromal heterogeneity, preserved IFN-responsive transcriptional programs, and extensive alternative splicing. Rather than acting as a passive marker of inflammation, PRELP reflects a specialized stromal niche linked to immune competence rather than acting as a simple marker of IFN activation. If validated in prospective cohorts, PRELP-centred transcriptional signatures may refine patient stratification and support the development of more robust immunotherapy strategies.

## Data Availability

The RNA sequencing data generated in the study are deposited in the NCBI Gene Expression Omnibus (GEO) repository, accession number GSE347202. Alternative-splicing datasets generated in this study through rMATS analysis are publicly available in Zenodo under the following record: https://doi.org/10.5281/zenodo.22077547. Additionally, original contributions presented in the study are included in the article/**Supplementary Material**. Further inquiries can be directed to the corresponding author.
