# Supplementary material for: PRELP-associated transcriptomic remodeling links alternative splicing and immune checkpoint blockade outcome in melanoma
Source: Front Immunol. 2026 Sep 15;17:1896231. doi: 10.3389/fimmu.2026.1896231 (PMC13621357; doi:10.3389/fimmu.2026.1896231)

Supplementary Figure 1: Sashimi plots showing significant exon-skipping events in PRELP-high and PRELP-low melanoma cells.

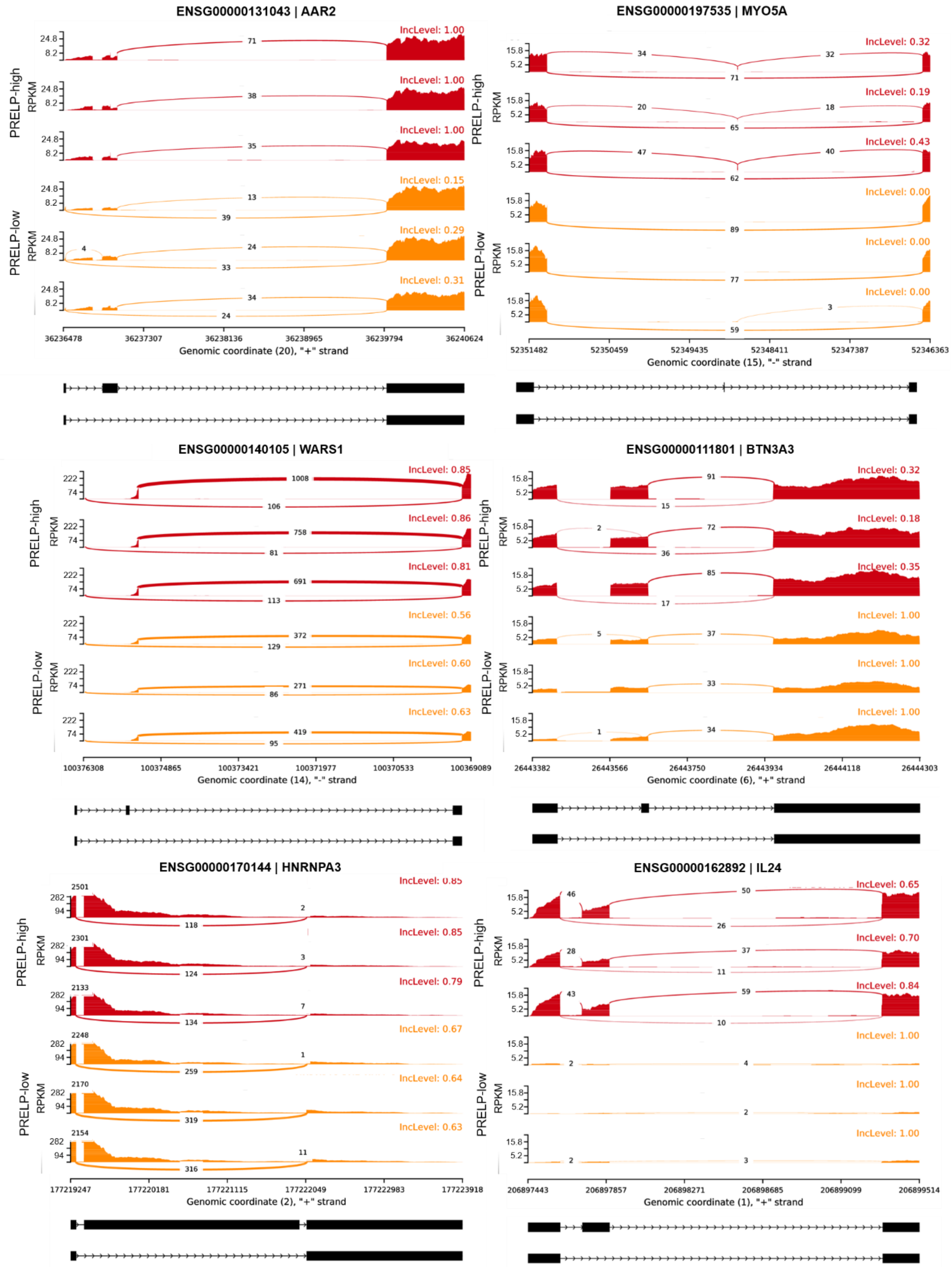

The upper panels display read coverage across the selected genomic region and splice-junction reads supporting alternative exon usage in PRELP-high and PRELP-low BUF1088 melanoma cells. Junction arcs represent exon–exon splice junctions, with arc labels indicating the number of supporting reads. The lower panel shows the corresponding transcript

annotation, including the exon-included isoform and the exon-skipped isoform generated by the differential splicing event.

Supplementary Figure 2: Validation of interferon-associated gene expression in PRELP-high and PRELP-low BUF1102 melanoma cells.
A: RT-qPCR validation of IRF7, IFI27, IFI44, and IFITM3 identified from RNA-seq analysis in PRELP-high and PRELP-low in BUF1102 melanoma cells.
B: RT-PCR validation of selected exon-skipping events in IFI27 and IL24 in BUF1102 melanoma cells.

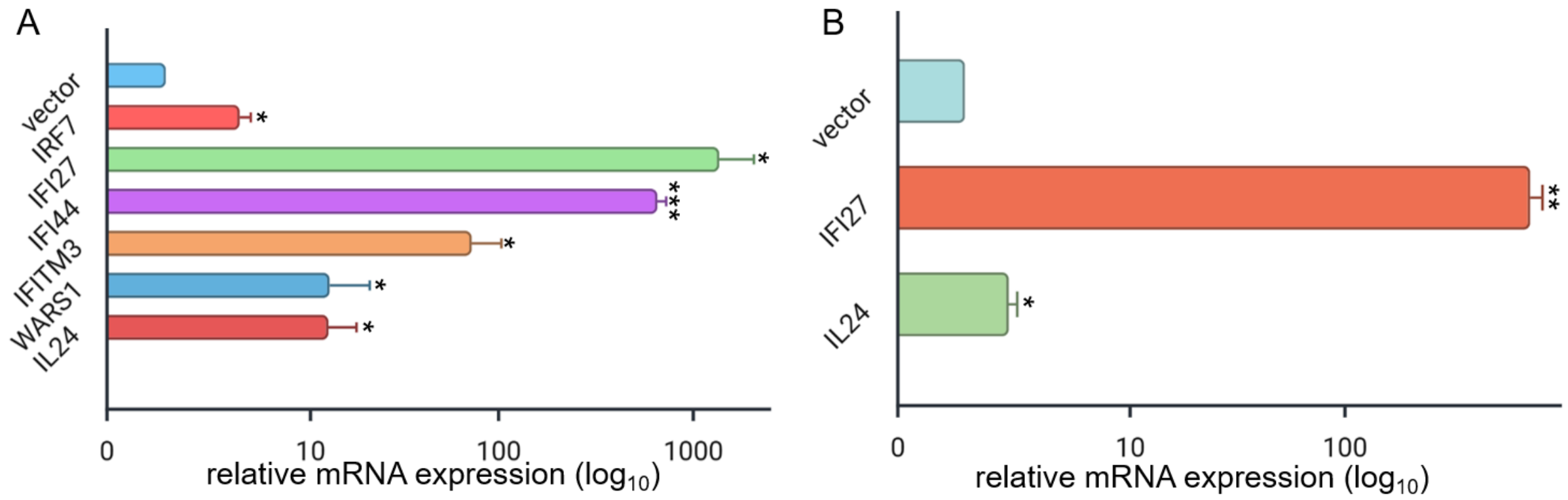

Supplementary Figure 3: Conditioned medium from PRELP-overexpressing BUF1088 melanoma cells induces interferon-associated gene expression in BJ fibroblasts. BJ fibroblasts were exposed to conditioned medium from vector-control or PRELP-overexpressing melanoma cells, and gene expression was analyzed by qPCR. Expression of (A) IFI27, (B) IRF7, (C) IFITM3, (D) WARS1 and (E) IL24, is presented as log2-transformed expression relative to untreated BJ control cells.

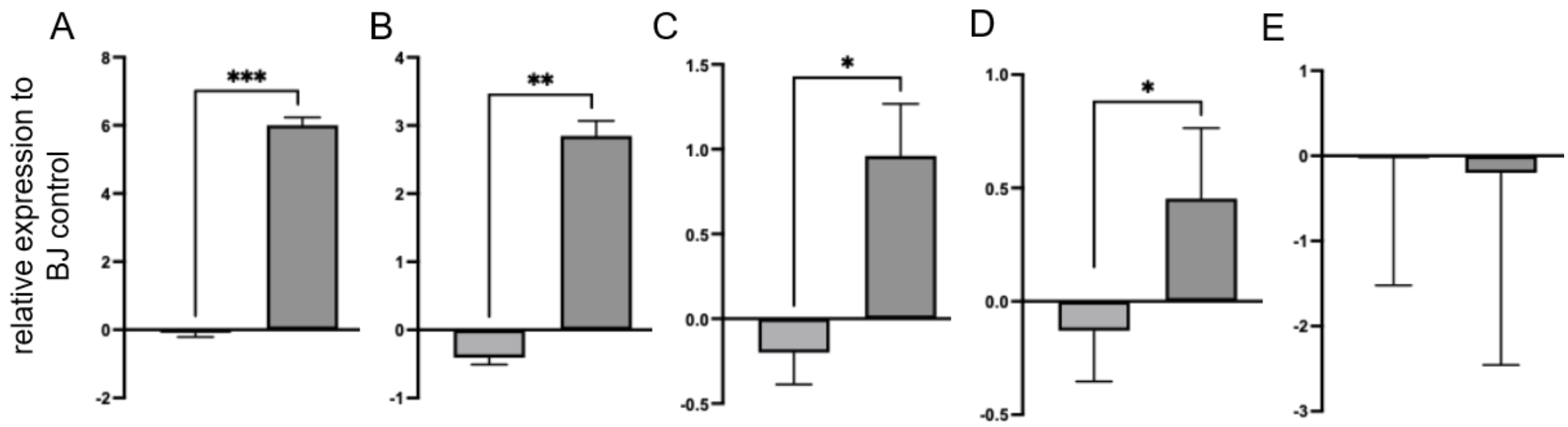

Supplementary Figure 4: DotPlot showing PRELP and PRELP-related gene signatures across annotated cell populations in the GSE115978 melanoma scRNA-seq dataset. Dot size represents the percentage of cells expressing each gene, and color intensity indicates scaled average expression. PRELP was enriched predominantly in CAFs, whereas IFN-related genes showed broader cell type-specific expression patterns across immune, endothelial, macrophage, malignant, and stromal compartments.

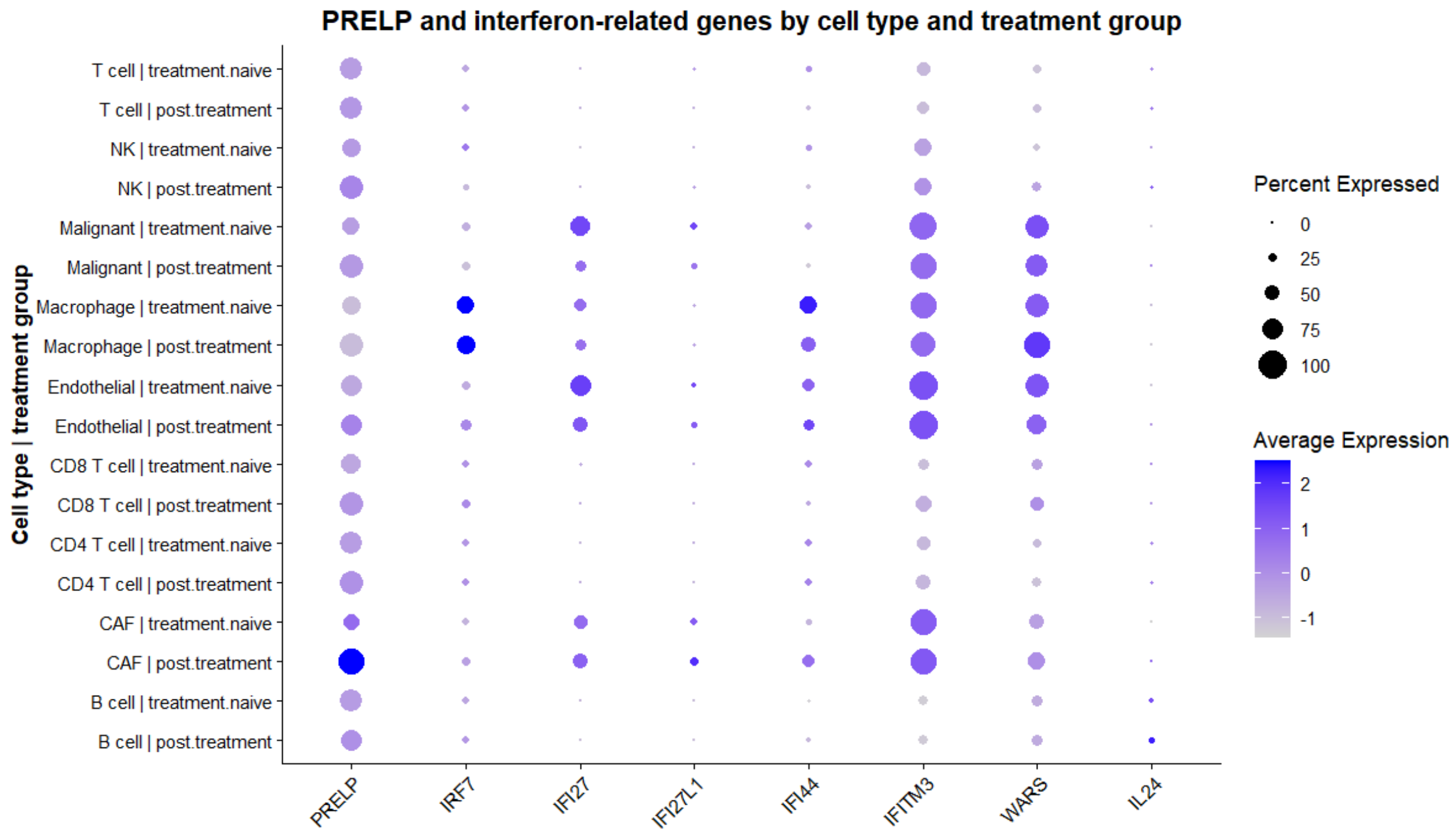

Supplementary Figure 5: UMAP visualization of PRELP expression in GSE72056 melanoma scRNA-seq. (A) annotated cell populations in the GSE72056 melanoma scRNA-seq dataset. (B) PRELP expression projected onto the same UMAP space.

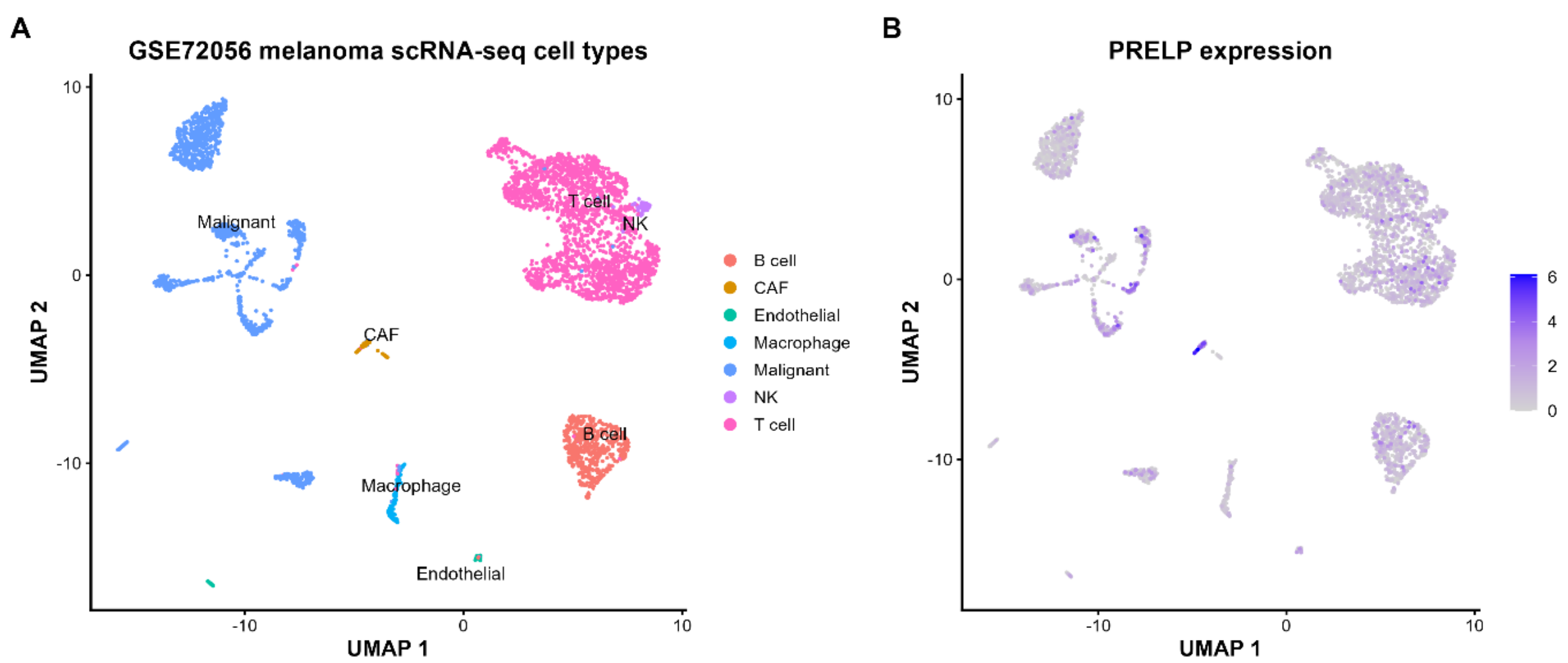

Supplementary Figure 6: UMAP plots of PRELP and PRELP-related gene signatures in GSE72056 melanoma scRNA-seq. Expression of PRELP, IRF7, IFI27, IFI27L1, IFI44, IFITM3, WARS/WARS1, and IL24 was projected onto the UMAP embedding of the GSE72056 melanoma single-cell dataset.

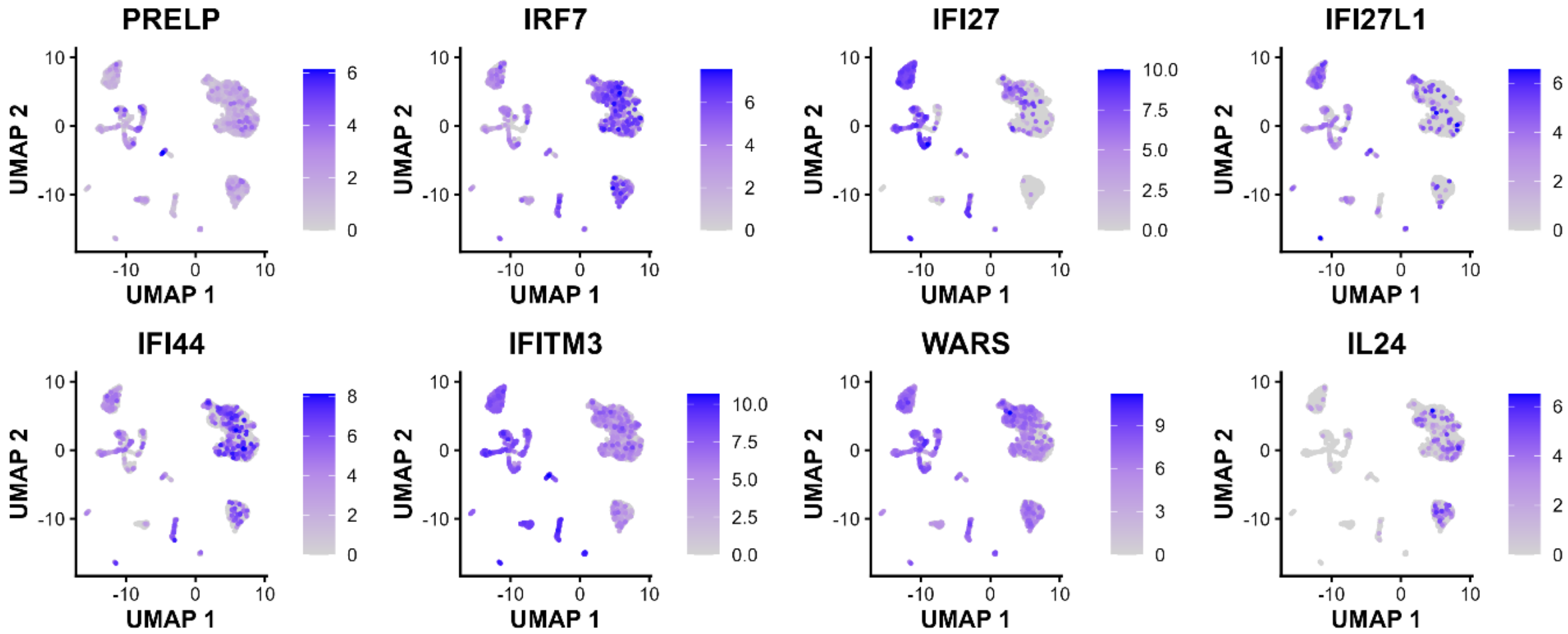

Supplement: Supplementary file 1 [file DataSheet1.pdf]
